# The Significance of the Entropic Measure of Time in Natural Sciences

**DOI:** 10.3390/e27040425

**Published:** 2025-04-14

**Authors:** Leonid M. Martyushev

**Affiliations:** 1 Technical Physics Department, Ural Federal University, 19 Mira St., 620002 Ekaterinburg, Russia; leonidmartyushev@gmail.com; 2 Institute of Industrial Ecology, Russian Academy of Sciences, 20 S. Kovalevskaya St., 620219 Ekaterinburg, Russia

**Keywords:** laws of development, maximum entropy production, universal law of growth, carcinogenesis, evolution

## Abstract

The review presents arguments emphasizing the importance of using the entropic measure of time (EMT) in the study of irreversible evolving systems. The possibilities of this measure for obtaining the laws of system evolution are shown. It is demonstrated that EMT provides a novel and unified perspective on the principle of maximum entropy production (MEPP), which is established in the physics of irreversible processes, as well as on the laws of growth and evolution proposed in biology. Essentially, for irreversible processes, the proposed approach allows, in a certain sense, to identify concepts such as the duration of existence, MEPP, and natural selection. EMT has been used to generalize prior results, indicating that the intrinsic time of a system is logarithmically dependent on extrinsic (Newtonian) time.

## 1. Introduction

Traditional methods of defining the metric of time are based on astronomical, mechanical, electromagnetic, and quantum regularities. These time metrics, according to modern understanding, are based on reversible phenomena and the measurement of periodicity. However, are such metrics suitable for describing irreversible processes that form the basis of our existence and the evolving world around us? Additionally, it is often assumed a priori that the flow of time is uniform. However, as early as H. Poincaré noted [1]: “*We have not a direct intuition of the equality of two intervals of time. The persons who believe they possess this intuition are dupes of an illusion. When I say, from noon to one the same time passes as from two to three, what meaning has this affirmation? The least reflection shows that by itself it has none at all. It will only have that which I choose to give it, by a definition which will certainly possess a certain degree of arbitrariness*”.

In light of these questions, the close relationship between entropy and time has been frequently discussed in the literature, raising the possibility of using entropy as a measure of time (see e.g., [2,3,4,5]). This idea is clearly illustrated by the following quotation from E. Mach (cited from [4], p. 145): “*If the entropy of the world could be determined, it would be an absolute measure of time and it would be, at best, nothing but a tautology to say that the entropy of the world increases with time*”.

The entropic measure of time (EMT) was most consistently and explicitly introduced in Ref. [6]. In this work, the change in time (Δ*τ*) and entropy production within the system (Δ*S*) are proportional to each other:

Δ*τ* ∝ Δ*S* (1)

Here, the entropy *S* is traditionally related to the logarithm of the number of possible states describing the system. In general, entropy is understood in the most general, informational sense, but for many practically significant cases, it can be regarded as thermodynamic entropy [6]. In this case, the entropy production can often be determined by measuring the amount of heat released by the system and its temperature. It is evident that the entropy measure of time (1) is meaningful only in the presence of irreversible processes within the system. In the case of equilibrium or for hypothetical (idealized) reversible processes within the system, EMT loses its meaning. The time defined by (1) will be referred to as internal time in this paper. This time characterizes the duration of irreversible processes within the system.

Metric (1) allows one to *quantitatively* describe two main characteristics of the time of a process: the duration of the process and the irreversible sequence of events in this process. Importantly, this metric does not a priori assume the uniformity of the flow of time. Furthermore, the metric (1) possesses three crucial qualities that distinguish it from traditional metrics [6]. Firstly, it is universal, since it can be introduced for any real system in which any changes occur. Secondly, such a metric allows, if necessary, to deductively obtain the laws by which the system develops. Thirdly, the metric of time has the property of relativity and subjectivity, which is important from the point of view of modern ideas initiated largely by the works of A. Einstein.

The work [6] and closely related Refs. [7,8,9] demonstrated that the introduction of the entropy-based metric of time leads to interesting implications for theoretical physics and developmental biology. This is the advantage of these works compared to previous works, in which the relationship between entropy and time was mostly discussed speculatively and philosophically, without any specific logical analysis of potential consequences. However, the ideas proposed and developed in Refs. [6,7,8,9] were not always consistent and failed to provide a complete picture or clear prospects for further development. As a result, they did not attract significant attention. In this regard, the objective of the present work is to provide a brief review of research related to the entropic time metric, to systematize existing studies, and to demonstrate how considering the duration of a non-equilibrium (irreversible) process based on (1) offers a novel perspective on several well-known theories.

## 2. The Laws of Physics and Cosmology

This section will demonstrate the potential of the entropic time metric in the context of establishing the laws of physics. This study is significantly influenced by the works of the outstanding astrophysicist E.A. Milne [10,11,12]. Let us dwell on two very original ideas of Milne’s works that are most important for the subsequent discussion.

The first idea concerns Milne’s perspective on the laws of nature. Milne argues, based on his results, that by selecting a set of kinematic properties of matter as axioms, we can deductively derive all the laws governing the world around us, from Newton’s laws to electromagnetism. As a result, the laws of nature become indistinguishable from theorems, and theoretical physics itself begins to resemble Euclidean geometry. In Milne’s view, the structure of the Universe entirely determines the physical laws that exist within it. Here is one of his quotes [12]: “*To begin with laws of nature and then add an arbitrary content on which the laws are to act is logically self-stultifying; for we have no guarantee that the laws assumed are compatible with the content assumed*”. For Milne, the most crucial initial step in constructing a theory is the choice of a time scale. Since, according to Milne, there is no natural measure of time, he introduced time in such a way that a certain class of observers move uniformly relative to one another. This method of timekeeping leads him to the concept of kinematic time, denoted as *t_m_*. Let us quote Milne [10]: “*There is no natural uniform scale of time, because we cannot say what we mean by the word uniform in relation to time; we cannot catch the fleeting minute and put it alongside a later minute. Sometimes it is said that a uniform scale of time is defined by a periodic phenomenon. But this is to beg the question… The system which has provided our frames of reference can be used to define a scale of time: if we can give a meaning to saying that the fundamental particles are all separating at constant velocities, this will give us a scale of time to work with.*” For Milne, the measure of length is secondary; it is introduced based on the observer’s perception of the passage of time and the transmission of light signals at a constant rate.

Milne’s second idea, which emerged from his theoretical analysis, suggests that physics uses not one but two different notions of time. One time, *t_m_* is used, for instance, in describing electromagnetic and atomic processes, while another time, *τ_m_* is applied in formulating the laws of Newtonian mechanics and gravity. Milne obtained a logarithmic relationship between these times:

*τ_m_* = *t*_0_ *ln*(*t_m_*/t_0_) + *t*_0_ (2)

where *t*_0_ is a normalization constant that represents the present age of the Universe on the *t_m_*-scale.

Following from (2), if the first-time scale *t_m_* is conventionally assumed to be uniform, then the second time scale *τ_m_* turns out to be non-uniform. Moreover, a finite time interval in the first scale corresponds to an infinite interval in the second time scale. One of the consequences of introducing such time scales was Milne’s conclusion about the time dependence of Planck’s constant and the gravitational constant (these results, as is known, later had a profound influence on the cosmological ideas of P. Dirac). For small time intervals (of the order of hundreds of years), the non-uniformity of the two time scales is extremely insignificant, which, according to Milne, is the reason why *t_m_* and *τ_m_* are identified in physics. However, when addressing astronomical problems, such as the age of star clusters and the Universe, accounting for this difference becomes crucial.

A discussion of modern cosmological problems from the perspective of Milne’s theory, as well as further developments of this theory, can be found in the works of Ram Vishwakarma [13,14,15]. According to him, even in its classical form, the Milne model significantly alleviates the so-called Hubble tension [15]. Cite an excerpt from his work [13]: “… *various cosmological observations can be explained satisfactorily in the framework of one such theory—the Milne model, without requiring the dark sectors of the standard approach. Moreover, the model evades the horizon, flatness and the cosmological constant problems afflicting the standard cosmology. Although Milne’s theory is an incomplete, phenomenological theory, and cannot be the final theory of gravitation, nevertheless, it would be worthwhile to study these coincidences, which may help us develop insight about the would-be final theory*”.

Despite the revolutionary nature of Milne’s ideas about time, certain points raise questions. First, the introduction of time based on certain requirements for the properties of clocks used by observers moving relative to one another appears to be just one possible approach, lacking a fundamental justification. Moreover, this approach does not reflect two essential properties of time—its universality and directionality. According to Milne, the distinction between the two time scales manifests experimentally only over very long time intervals and is significant only in physics. Secondly, Milne’s rejection of the fundamentality of the measure of length and its replacement by a measure of time, along with the additional assumption about the constancy of the speed of light, cannot be considered convincing, as other constants then turn out to be time-dependent.

In this regard, in the works [8,9], an attempt was made to obtain the law of development and interaction using other postulates. In these works, the measurement standard of length is introduced as the size of a perfectly rigid body (or as a certain number of wavelengths of a certain emission line of some atom), while the measure of time is introduced using (1). In this formulation, the measures of length and time are not as strictly mathematically coupled as assumed in Milne’s theory and in many other modern theories. In the Refs. [8,9], the method of introducing and determining entropy defines the degree of coupling between the measures of length and time, which may vary within certain limits. By considering the simplest model of *N* particles occupying some space volume of the size *G*, a kinematic law was obtained that relates these quantities and time *τ*. Below are the most interesting consequences of this law. The first consequence: As time increases, the system undergoes accelerated expansion. If *G* remains constant, the number of particles decreases. The second consequence: As *τ* increases, analogs of repulsive and attractive forces emerge in the system. For relatively dense systems, these forces are inversely proportional to the square of the system’s size, while for sufficiently rarefied systems, they have constant values. The third consequence: The rate of increase in the system’s size is proportional to its size. The fourth consequence: If it is assumed that there exists an extrinsic time *t* relative to which *G* increases at a constant rate, then for sufficiently large times, the entropic time *τ* and *t* are related as:

*τ* ∝ *ln t* (3)

According to Equations (2) and (3), the relationship between the times *τ_m_*, *τ* and the time for which the size of the system under consideration changes at a constant rate turns out to be similar. This is particularly interesting because *τ_m_* is introduced by Milne at the final stage of his constructed theory to ensure that the obtained law of interaction between particles corresponds to the classical law of gravitation [10,11,12]. In contrast, *τ* is introduced in Refs. [8,9] based on an entropic measure and forms the foundation of the theory being developed, from which the inverse-square law of interaction directly follows. Thus, in Milne’s theory, the most fundamental time is *t_m_*, and the observer uses *τ_m_* to formulate the laws of mechanics in the form that has historically been established. On the contrary, according to Refs. [8,9], the most fundamental time is *τ*, measured by the entropic measure, while *t* is introduced purely mathematically based on the hypothetical assumption of the existence of a time measure in which the boundary of the system under consideration moves uniformly (or, in other words, existence of some "uniform", Newtonian time). Determining which of these approaches—Milne’s [10,11,12] or that proposed in Refs. [8,9]—provides a more consistent and promising foundation for theoretical development remains an important subject for future research.

Let us briefly discuss a number of works that address issues somewhat related to those outlined above.

1. First and foremost, it is essential to mention works [16,17]. In these studies, classical gravity and the equations of general relativity are derived by calculating the change in entropy of a certain surface (a holographic screen). Here, gravity is considered not as a fundamental interaction, but as an emergent phenomenon (an entropic force) that arises from the statistical behavior of microscopic degrees of freedom encoded on a holographic screen. The works [16,17] laid the foundation for the development of a very interesting and fruitful alternative to the standard cosmological paradigm, which has numerous followers (see, for example, [18]). A common feature of two theories [16,17] and [8,9] is the conclusion about the connection between entropy and gravity. However, these theories differ fundamentally in their treatment of the role of time. In Refs. [16,17], as in Einstein’s theory, time is not distinguished from spatial variables in any fundamental way. In contrast, in the second theory [8,9], time is considered a special variable, uniquely linked to entropy, as opposed to space.
2. Caticha’s entropic time [19,20]. Dynamical laws (e.g., quantum theory) are derived as an application of entropic methods of inference. This approach is a development of the classical and well-known works of E. T. Jaynes [21]. A theory is constructed by maximizing relative entropy subjected to constraints that reflect the information relevant to the problem at hand. A. Caticha introduced entropic time as a book-keeping device designed to keep track of the accumulation of change. He defined time in a special way using equation for transition probability (formally identical to the Chapman–Kolmogorov equation), rather than directly through entropy change, as defined in Refs. [8,9]. A dynamic driven by entropy naturally leads to an ‘entropic’ notion of time. Time itself is treated as an additional assumption, ensuring that the duration of time is defined so that the resulting dynamics appear simple [20]. An important quote from [20] states:

“*The derivation of laws of physics as examples of inference led us to introduce the informationally motivated notion of entropic time, which includes assumptions about the concepts of instant, simultaneity, ordering, and duration. It is clear that entropic time is useful, but is this the actual, real, “physical” time? The answer is yes. By deriving the Schrödinger equation (from which we can obtain the classical limit) we have shown that the t that appears in the laws of physics is entropic time. Since these are the equations that we routinely use to design and calibrate our clocks, we conclude that what clocks measure is entropic time. No notion of time that is in any way deeper or more “physical” is needed.*”

Another important quote by Caticha is also relevant [19]: “*The asymmetry is the inevitable consequence of entropic inference. From the point of view of entropic dynamics, the challenge does not consist in explaining the arrow of time, but rather in explaining how it comes about that despite the arrow of time some laws of physics turn out to be reversible. Indeed, even when the derived laws of physics—in our case, the Schrödinger equation—turns out to be fully time-reversible, entropic time itself only flows forward*.” The author, as he himself writes, is a proponent of J. Barbour’s ideas [22,23] on the relational notion of time, and this is evident from the quote above. Entropic time (or its metric) does not claim to explain big questions (such as the existence of time or its asymmetry), but helps in obtaining the laws of description of the surrounding world.

Despite some similarities between the ideas described here and those in [8,9], a fundamental difference lies in the role of entropy and time. In Caticha’s theory, the maximization of relative entropy serves as the foundation for constructing dynamics, with time acting as an auxiliary concept. In contrast, in [8,9], dynamics is directly built upon a time measure based on standard (rather than relative) entropy, and the maximization of this entropy is not a necessary requirement.

3. P. Magain and C. Hauret proposed an explanation for the accelerated expansion of the Universe without dark energy by assuming that the cosmological time of observers is proportional to the entropy of the region of the Universe that is causally connected to them [24,25]. They propose the existence of two different times: (1) the coordinate (conventional) time *t*, which is assumed to flow at a constant rate along the evolution of our universe; (2) the cosmological time *τ*, which is assumed to depend on the state of the universe and to control all physical processes. Every measurement is made in time *τ* (we live in this time). Cosmological time is not uniform, and it is linked to the entropy of the photon gas of the cosmic microwave background. Using this connection, Magain and Hauret proposed determining *τ* by recalculating the coordinate time *t* present in the Robertson–Walker metric and the Einstein equations.

The works [24,25] resonate with Milne’s work, as they suggested considering two times when analyzing processes in the Universe. Additionally, the studies [24,25] can be considered close to [6,7,8,9] due to the proposed connection between time and the entropy of an evolving process. A significant drawback of Magain and Hauret’s work compared to Milne’s is that their second time does not emerge as a consequence of a unified logical approach in constructing physics but rather as an auxiliary assumption that helps interpret the results of the accelerated expansion of the Universe. Compared to [8,9], another drawback of Magain and Hauret’s work is the lack of confidence in the universality and significance of the entropic metric they used for *τ*, which they treated more as a fortunate intellectual discovery (a trick) to rid cosmology of dark energy. In this regard, it is appropriate to cite [25]: “*We nevertheless want to stress that—as pointed out earlier—other mathematical dependences between time and entropy or any other parameter characterising the dynamical state of the universe might be proposed or even better derived*.”

## 3. Maximum Entropy Production Principle

The earliest formulations of this principle are presented in works [26,27,28,29]. An overview of current research concerning the Maximum Entropy Production Principle (MEPP) is provided in Refs. [30,31]. These works demonstrate that MEPP is a fundamental principle underlying non-equilibrium thermodynamics and statistical physics, and it also has very broad applications in materials science, chemical kinetics, climate science, and biology.

The modern formulation of MEPP is as follows [31]: *at each hierarchical level of the evolution, with preset external constraints, a relationship between the cause and the response of a non-equilibrium system is established in order to maximize the entropy production density.* When considering the hierarchical level traditionally described by classical non-equilibrium thermodynamics, the cause and the response are understood, respectively, as thermodynamic forces and fluxes and the entropy production density is understood as a term in the balance law for a continuous system, which is associated with the production of entropy per unit volume within the system [28,29,30,31].

Like any fundamental principle (such as Fermat’s principle in optics or the principle of least action in mechanics), MEPP is formulated based on the generalization of existing experimental and theoretical data. As a result, the principle becomes a statement that most efficiently and briefly summarizes the existing experience, and from which this experience can be deductively inferred. For this reason, principles are not proven. At the same time, it is always important to identify connections between various principles and fundamental concepts that exist in science.

In this regard, let us discuss MEPP from the perspective of EMT. Consider the evolution of a non-equilibrium system at some hierarchical level with preset external constraints. Suppose that, for this system, several relationships between the cause and the response are possible. Consequently, each *i*-th relationship corresponds to a certain entropy production Δ*S_i_*. According to (1), the system with the highest entropy production among all Δ*S_i_* will have the longest duration of existence. As a consequence, an observer using EMT should conclude that the system maximizing entropy production is the most viable. This situation resembles the survivorship effect: under preset external constraints, the "surviving" system will always be the one with the highest entropy production. From the EMT point of view, MEPP turns into tautology, similar to the example by E. Mach quoted in the Introduction “…*entropy of the world increases with time*”. This is truly remarkable, considering how many insights, meticulous studies, and debates among experts over nearly eighty years were required to formulate and establish MEPP as a fundamental principle [30,31].

It is well known that one of the consequences of MEPP is that non-equilibrium systems with equal entropy production will coexist [30,31]. These non-equilibrium systems are assumed to have emerged approximately simultaneously under the same external conditions, although these systems could, in general, differ from each other, for example, morphologically. According to EMT, such non-equilibrium systems with equal entropy production should have the same duration of existence. However, the requirement that coexisting systems must have the same duration of existence again turns into a tautology. It is important to note that observers associated with such coexisting systems and using EMT will possess similar measures of time. If these observers derive laws of evolution based on these measures (for example, as performed in Section 2), these laws will turn out to be similar. In this regard, one can speak of a coincidence of laws for observers belonging to different coexisting systems. This statement can be considered an important and interesting generalization of the Galilean–Einstein principle of relativity.

As demonstrated in Section 2, the entropic time measure provides a new perspective on the laws of quantum mechanics and gravity. At the same time, the MEPP (which has been shown to be directly related to the entropic measure) is also considered a deductive foundation for deriving fundamental equations (models) in non-equilibrium statistical physics. For instance, in [32], the principle is reformulated in a more mathematically rigorous and convenient manner, according to the author, and it is shown how MEPP is directly connected to the majority of currently used equations: both widely known ones (statistical models of relaxation, kinetic models for the Boltzmann equation, equations of macroscopic non-equilibrium thermodynamics, chemical kinetics, etc.) and more specialized models (continuum mechanics with fluctuations, quantum statistical mechanics, quantum thermodynamics, etc.). MEPP also plays a crucial role in considering the most fundamental cosmological problems. For example, in Ref. [33], it is argued that, from the perspective of observers, physical parameters are most likely to be found in the range of values for which the total entropy production within a causally connected region is maximized. The author refers to this statement as the causal entropic principle and uses it to compute the expected value of the cosmological constant in our universe. Despite the absence of more explicit anthropic criteria, the result turns out to be in excellent agreement with the observation.

## 4. Growth in Non-Living and Living Nature

The introduction of the entropic measure of time and MEPP provides a unified perspective on development in both non-living and living nature, particularly regarding the selection and the coexistence of different growth forms. In this section, we will also demonstrate the possibility of deriving a universal equation to describe growth curves and, based on this, establish a connection between EMT and the extrinsic time with respect to the growing system.

### 4.1. Entropy Production and MEPP

Growth in nature is a typical irreversible process and, as a consequence, has a non-zero entropy production. In this process, an increase in the mass Δ*m* of a certain system occurs due to the transformation of a substance from the environment. The most typical ways of such a transformation are single or multiple chemical reactions and other similar processes, such as crystallization, condensation, vaporization, etc. Due to the spontaneity of this process, the transformed substance passes from a state with a higher value of chemical potential to a state with a lower chemical potential. Let us make the simplest assumption that the difference in chemical potential between the environment and the growing system Δ*μ* remains constant. In this case, the entropy production during growth can be written as [28,34]:

Δ*S* ∝ Δ*μ* Δ*m* (4)

The most well-known example of growth in non-living nature is the formation of crystals. Let us consider this process. Numerous experimental and theoretical studies related to crystallization from solution/vapor or solidification from a melt reveal that non-equilibrium crystal growth occurs with the maximization of entropy production. A review of these studies is presented, for example, in Refs. [30,31]. Following MEPP, especially, is clearly observed during morphological phase transitions as a result of which one non-equilibrium growth form discontinuously transits into another. In such cases, it has been found that the growth form with the maximum entropy production is observed with the highest probability. It took several decades to verify and substantiate this result [30,31]. However, from the perspective of the entropic measure of time discussed here, this conclusion appears more than obvious: The greatest entropy production, according to Equation (1), is identical to the longest duration of structure growth and, consequently, the highest probability of observing this structure. The latter is true if the moment of occurrence of morphological phase transitions is random in time.

In addition to non-equilibrium crystallization, conclusions regarding the selection of viable structures based on MEPP have also been drawn in studies of the growth and evolution of viscous fingers resulting from the pressure-driven displacement of one immiscible fluid by another in a Hele–Shaw cell, as well as in the growth of vapor bubbles during boiling [31]. Thus, in these cases, the application of EMT would also be useful. This conclusion naturally extends to biological systems, which are discussed below.

The behavior of entropy production during growth and development in biological systems has been studied for quite a long time (see, for example, the reviews presented in Refs. [35,36,37]). The determination of this quantity is typically associated with measuring or calculating either the heat released from surfaces with known temperatures or oxygen consumption. Interest in this topic arose after the publication of the works of I. Prigogine and J. Wiame [38]. According to their hypothesis, during the growth and development of organisms, there is a continuous decrease in entropy production. There is extensive experimental evidence supporting this behavior. However, in the case of early-stage development of living systems (particularly embryonic and initial post-embryonic development) and the growth of microbial cultures, entropy production, on the contrary, increases. The consideration of growth in biological systems from the perspective of MEPP is practically absent in the literature. This is because verifying or applying MEPP requires the presence of alternatives in development. Indeed, a simple decrease or increase in entropy production during growth says nothing about this principle, since the maximum entropy production must be selected from several possible options under the given conditions. As is known, ontogenesis is characterized not only by irreversibility, unevenness, and adaptability, but also by the largely programmed nature of the processes taking place. It is this last feature that complicates the analysis of biological growth from the perspective of MEPP and requires the development of special experiments in the future. However, there is one example in ontogenesis that highlights the importance and potential of MEPP: carcinogenesis. Since this process is rarely discussed in the literature from the perspective of MEPP, we will examine it in more detail.

The study of carcinogenesis from the perspective of non-equilibrium thermodynamics, including specific calculations of entropy production and proposals for therapy, appears to have begun relatively recently, around 2004–2006 [39,40,41]. In these works, L.F. Luo and co-authors provided estimates of entropy production in normal and malignant cells, considering four main contributions: thermal, diffusion, chemical, and viscous effects [39,40,41]. Additionally, they examined cases involving external electromagnetic and ultrasound exposure on cells. The main conclusions were that the largest contribution to entropy production comes from chemical processes, which amount to approximately 10^−16^ W/K (or of the order of 10^−2^ W/kgK) for malignant cells, and roughly half that for normal cells. According to the study’s estimates, if cells are exposed to electric pulses (~10 V/cm) or low-intensity ultrasound (<1 W/cm^2^, <1 MHz), the entropy production of normal cells can be made greater than that of malignant cells, which is highly significant for cancer treatment. In the study [42], various methods for increasing entropy production in normal cells for therapeutic purposes are discussed. Between 2012 and 2015, L.F. Luo and co-authors moved toward experimental verification of their previous findings [43,44,45,46]. They proposed an original method for measuring entropy production in living cells, which involved heating samples using a varying electric field and recording the released heat. Entropy production was measured in both normal and cancerous cells. It was found that with increasing field strength, entropy production in cancerous cells increased monotonically linearly, whereas in normal cells, it exhibited significantly nonlinear behavior. As a result, entropy production in healthy cells is greater than entropy production in cancer cells when exposed to an electric field in the range from 5 to 25 V/cm.

To summarize, L. Luo and colleagues repeatedly emphasized that for cancer treatment, it is necessary to achieve via external influences greater entropy production of healthy cells than that of cancer cells. In addition to medical data supporting this hypothesis, these researchers do not provide convincing modern scientific justifications for the effectiveness of such therapy. However, the effect that was discovered here has a simple explanation based on MEPP. Indeed, according to this principle, the most stable state of a non-equilibrium biochemical system is the state with maximum entropy production. Initially, the cells of a given organ coexist with approximately the same entropy production. However, due to aging, mutations, and other factors, at a certain stage of development, cells with higher entropy production than the majority of "healthy" cells emerge in the organism. The growth of these new (cancerous) cells represents a more favorable and stable direction of development from the perspective of MEPP. As a result, the non-equilibrium system (tissue, organ) “switches”: cancer cells begin their uncontrolled proliferation, suppressing normal cells, which have become thermodynamically unfavorable (as they locally produce less entropy than the new formations). The reversal (cure) of such a system is only possible by either reducing the entropy production of cancerous cells or increasing the entropy production of healthy cells. Only such therapy provides the previous stability of the non-equilibrium system of normal cells. This MEPP-based interpretation suggests that the robustness of carcinogenesis has a fundamental cause. It is worth noting that in the purely biological literature, there is no clear understanding of the nature of cancer robustness [47].

J. Nieto-Villar and colleagues also associate cancer robustness with the magnitude of entropy production, briefly mentioning MEPP in their latest work as a justification [48,49,50]. According to J. Nieto-Villar, the entropy production rate is a crucial parameter that indicates the possible behavior of a tumor. Based on the magnitude of entropy production, 10 key reactions out of 20 regulating the glycolysis process in a specific type of cancer cells were identified. These reactions, having the highest entropy production, should be the main targets in cancer therapy [50]. It was found that during glycolysis in hypoxic conditions, entropy production in cancer cells is higher than in normoxia, making tumors more robust under hypoxia. As a result, any form of therapy should be conducted under normoxic conditions [50]. Additionally, it was found that as intracellular pH increases (from 6.2 to 7.4), entropy production increases linearly. In studies [51,52], carcinogenesis is also discussed from the perspective of non-equilibrium thermodynamics, calculation of entropy production, its extremal properties, and possible methods of influencing entropy production for cancer therapy. Overall, the results of these studies align with the discussions presented above.

In conclusion, it should be particularly emphasized that carcinogenesis is an extremely complex and multifaceted pathological process in the organism. The discovered universal laws underlying this process, apparently, should help in the search for universal approaches to the prevention and control of this disease. However, the example of the Warburg effect, which is a universal marker of cancer cells compared to normal cells, shows that more than 100 years of research into this phenomenon has not helped in the development of universal cancer therapies. The use of a balanced approach to cancer therapy that takes into account both its universal properties and the numerous specific features of different types of cancer is necessary.

### 4.2. Time and the Universal Law of Growth

As is well known, extrinsic time (also referred to as chronological, astronomical, or clock time) *t* often proves to be an inconvenient metric for analyzing growth in biology (see, for example, Refs. [53,54,55,56,57,58,59,60,61]). This is due to the fact that the duration of any period of development, even among genetically close species, can differ significantly in units of *t*. Factors such as body temperature, environmental conditions during growth, and the mass of the growing body influence these variations [58,59]. As a result, identifying any laws of development becomes very difficult. A convenient and universal time metric, associated with the internal processes in the organism, could reveal common growth patterns and laws that remain obscured when using traditional astronomical time. The search for such a metric has been ongoing for a long time [53,54,55,56,57,58,59,60]. This metric is referred to as intrinsic time *τ* (also known as physiological, developmental, or biological time). Metrics related to the organism’s metabolism are considered the most promising [53,55,56,57,60,61]. For instance, in Ref. [60], a time metric based on mass-specific metabolic rate is proposed. It is well-known that this rate is directly related to the entropy production density (normalized per unit volume or mass). Thus, in developmental biology, the idea of the necessity and importance of an entropic measure of time, as expressed in Equation (1), is independently emerging.

According to Equations (1) and (4), in the case of growth, the entropic time measure for a unit mass *m* can be written, up to a constant factor, as:

(5) $$d\tau \;\propto \frac{dm}{m}$$

As can be seen, to find the connection between EMT and extrinsic time *t* (which we will assume to be traditionally uniform, Newtonian), it is necessary to find the dependence of d*m*/*m* on *t* during growth. In the derivation, we will adhere to the hypothesis that growth is a universal phenomenon. Consequently, the growth law describing the mass evolution over time for various objects of both living and non-living nature should have the same functional form. It is important to note that this refers to the universality of the phenomenological dependence of *m* on *t*, while the microscopic mechanisms and governing laws of growth often differ significantly. A similar approach has been previously implemented in [7,62], which we will reproduce. The quantity m^−1^ d*m*/dt has the dimension of inverse time. Therefore, one can write the equation as:

(6) $$\frac{1}{m}\frac{dm}{dt}=\frac{a}{t}$$

where *a* is a certain dimensionless constant.

Equation (6) is not only the simplest possible growth equation, but also the only correct one based on the accepted hypothesis. Indeed, growth equations for various objects (crystals, plants, animals, etc.), as well as for objects at different levels of organization (parts of a crystal, plant organs, etc.), should be similar. Thus, this universal equation should not contain dimensional, time-dependent constants or variables specific to a particular growing object. For example, for plants, important dimensional quantities could be the wavelength of incident radiation and Planck’s constant; for a crystal, diffusion coefficients in the medium and the kinetic coefficient of crystallization, etc. Dimensional quantities that are crucial for the growth for one type of system may turn out to be only secondary or meaningless when considering the growth for other systems. Therefore, the hypothesis of universality necessitates the exclusion of all quantities that, in each particular case, could determine the growth rate. The only quantity with the dimension of "time" that is common to any growth process is age, *t*. It is important to note that the hypothesis of universal growth also implies that this growth is associated exclusively with growth in an environment that remains unchanged over time. Otherwise, the introduction of any time-dependent constants specific to each growth process would be required.

The equation coinciding with (6) was proposed between 1925 and 1935 by I. Schmalhausen based on empirical studies on the growth of chicken embryos and later on a number of other biological species in the context of embryonic and post-embryonic growth [63,64,65,66,67]. According to Schmalhausen, the power-law dependence of mass on time (*m∝ t ^a^*), modeled after (6), provides a significantly better agreement with observed data compared to the frequently used exponential growth formulas. Schmalhausen’s conclusions were also experimentally confirmed by other researchers (see, for example, [68,69]). Depending on the growing organism under consideration, values for the coefficient *a* were found to range from 0.1 to 10. Schmalhausen associated his power-law empirical model with the role of differentiated cells and their age-dependent numbers. In his opinion, exponential growth is observed only in organisms without differentiation (e.g., bacteria), while power-law growth occurs in organisms with differentiation (e.g., vertebrates). In our opinion, a dependence similar to Equation (6) has a crucial logical advantage compared to the exponential model: in the case of model (6), the specific growth rate is initially undefined (singular), in contrast to the commonly accepted exponential models. Indeed, a non-existent object cannot possess any rate. Rate is an emergent characteristic of the growth process; this is the result of the interaction between the environment and a fundamentally unique complex object arising within it.

In deriving (6), mass was chosen as the primary characteristic of growth. However, growth can also be characterized, for example, by volume or some other scale factor of length. The choice of a particular quantity depends on convenience or established tradition in a given field of research. It is interesting to note that if the expansion of the Universe is considered analogous to growth, and the scale factor is used instead of mass, then Equation (6) resembles Hubble’s law, where the Hubble parameter is taken to be *a/t* [18]. The connection of (6) with EMT, which will be discussed below, along with the analysis already presented in Section 2, suggests that such an analogy may not be coincidental.

Equation (6) can be generalized to a case where changes in the environment may occur as a result of the growth itself. The most obvious and simplest assumption is that these changes are proportional only to the mass of the growing body. As a result, (6) can be rewritten in the form [7,62]:

(7) $$\frac{1}{m}\frac{dm}{dt}=\frac{a}{t}-b$$

where *b* is a positive constant with the dimension of inverse time.

According to Refs. [7,62], Equation (7) describes growth over time well for both the initial and intermediate and final stages, being no worse than the generally accepted growth models: Gompertz, Verhulst, and Bertalanffy. In this case, Equation (7) was tested in the analysis of experimental growth curves both for the living organisms and for the growth of crystals.

According to Equations (5) and (6), the relationship between EMT and extrinsic time is given as *τ ∝ ln t.* This relationship coincides with Equation (2) and Equation (3), which were derived earlier based on entirely different assumptions. Moreover, a similar logarithmic relationship between times was proposed by G. Backman in 1943 based on extensive studies of plant and animal growth [70]. In his work, *τ* was considered exclusively as a measure of biological development, whereas *t* was treated as astronomical time. It is also worth noting that the obtained logarithmic law is reminiscent of the Weber–Fechner law, which states that subjective sensation is proportional to the logarithm of the stimulus intensity. In our case, intrinsic time (which can, in a sense, be called subjective mental time) is the logarithm of extrinsic time (stimulus). A similar “extrapolation” of the Weber–Fechner law, where physical stimulus is understood as not only light, sound, etc., but also temporary “impact”, can be found in the work [71].

According to Equations (5) and (7), the relationship between EMT and extrinsic time can be derived for growth occurring under non-constant external conditions [7]:

(8) τ ∝ a lnt−bt

Equation (8), in contrast to Equation (3), is valid only if the properties of the environment change under the influence of growth in direct proportion to the changing mass of the object [7].

In conclusion, we emphasize that the introduction and application of entropic time in the study of growth processes is a highly logical step. Indeed, a new complex system arises in an emergent, discontinuous manner, thereby breaking the uniformity of the spatial and temporal environment both within and near this system. The irreversible development and growth of the newly formed system maintain this spatial and temporal asymmetry, amplifying or attenuating it at different stages of growth. Thus, entropic time is born and exists during the emergence and growth of a system. It is an inherent property of the system, and therefore, it is most natural to study the properties of a growing system (regardless of its nature) using this form of time. Uniform and reversible time, external to the system, cannot be a natural measure. In such an external basis chosen for description, the laws of development of a growing system appear in a more complex and confusing form. This was noticed by biologists studying development long ago and led them to introduce the so-called biological time. However, as presented above, such biological time is often a special case of EMT. Therefore, there is no need to distinguish biological objects from others. The regularities of growth, as well as the concept of time itself, are universal.

## 5. Evolution of the Surrounding World: An Entropic Perspective

The study of evolution from perspectives related to entropy and MEPP has been undertaken by numerous researchers, with the origins of this field tracing back to over a century. A well-known statement by L. Boltzmann (1886) illustrates this: “*The general struggle for existence of animate beings is therefore not a struggle for raw materials—these, for organisms, are air, water and soil, all abundantly available—nor for energy which exists in plenty in any body in the form of heat (albeit unfortunately not transformable), but a struggle for entropy, which becomes available through the transition of energy from the hot Sun to the cold Earth”* [72]. For an introduction to the problem about the connection between MEPP and evolution (primarily biological evolution), review papers [30,31,73,74,75,76,77,78,79,80,81,82,83,84,85] can be recommended. Let us make a brief summary of these works. According to MEPP, at each hierarchical level, a non-equilibrium system will, through self-organization, choose the state that maximizes the density of entropy production (in many cases, we can speak not only of entropy production but also of heat production). As a result, the formation of Earth and the emergence of life on it, the increasing complexity of living beings throughout evolution, the appearance of humans, and the entire development of our civilization can be considered as a step-by-step process following MEPP. The results of works [75,76,79,80,82,86,87,88,89] support this conclusion. MEPP proves to be a crucial principle explaining the often-observed directionality (progressiveness) of evolution, both biological and technological. The perspective on evolution from the standpoint of MEPP provides the opportunity to give the first quantitative definition of life [31]. According to Ref. [31], life is defined as a region of space–time with specific entropy production ranging from 10^3^ to 10^5^ times the specific entropy production of a star close to where this region is located (here, specific entropy production is calculated per unit volume, and for the Sun, it is approximately 10⁻^4^ W/(m^3^ K)).

As is known, the first consistent evolutionary theory was proposed by J. Lamarck in 1809 [90]. The foundation of this theory was Lamarck’s idea of an intrinsic striving for perfection (*Le pouvoir de la vie*) inherent in all living beings. According to Lamarck, this striving determines the observed tendency to increase the organization of living beings. At present, MEPP can be considered as such a guiding force. It is important to emphasize that, according to MEPP, not only living beings evolve, but also any other non-equilibrium processes. Consequently, the need for Lamarck’s mysterious force of nature, which acts exclusively on living entities, becomes unnecessary. Darwin’s theory, proposed in 1858, remains the foundation of modern concepts of biological evolution [91]. The key difference between this theory and the previous one was the replacement of the postulate of an intrinsic striving for perfection with the principle of natural selection. According to this principle, the number of individuals with higher adaptation to environmental conditions (i.e., those possessing the most favorable traits) increases in a population, while the number of individuals with unfavorable traits decreases. This is the essence of the so-called struggle for existence and, as L. Boltzmann correctly noted (see the quote above), this struggle occurs for entropy (resources containing low entropy, to be more precise). In this struggle, it is not only the quantity of resources consumed but also the time during which these resources are processed for the benefit of the organism that is evidently important. The faster an organism processes available resources, the more entropy it produces per unit of time. Such organisms are preferable from the point of view of MEPP. These organisms leave behind more offspring, thereby further increasing entropy production. A type of chain reaction arises in the form of positive feedback that continuously increases entropy production. Thus, MEPP offers a unique way to reconcile Lamarck’s idea of “striving toward perfection” with Darwin’s concept of natural selection. Emerging and developing through the “struggle for existence” in accordance with MEPP, organisms often (though not always) become more complex, since greater organization and complexity require the consumption of greater free energy, which, when processed, releases greater entropy [31].

Why is only the increase in entropy production preferable in the struggle for existence? Indeed, consider two organisms that consume the same amount of resources per unit of time, but one of them processes the available energy more efficiently. As a result, this organism has more energy for its development and reproduction while generating less entropy. Would the evolution of such an organism not contradict MEPP? There are two objections here. First, the effect of increasing efficiency in real systems is not significant. Therefore, the evolution of an organism is largely determined by its ability to expand available resources and explore new ecological niches, rather than stagnate at the achieved level and optimize existing consumption mechanisms [92,93,94,95]. It is precisely expansion, not optimization, that leads to significant changes and the abrupt emergence of new traits, which in turn increase entropy production. In this regard, the situation is very close to that which arises when considering the relationship between MEPP and Prigogine’s principle, the first of which is associated with the emergence of new qualities, and the second with the optimization of what has been achieved [30,31,73]. Secondly, following a strategy of efficient resource use will not ultimately lead to a reduction in entropy production. Here, it is appropriate to recall the so-called Jevons Paradox: technological improvements that increase energy efficiency do not lead to a decrease but rather to an increase in energy consumption [96,97,98,99]. This occurs because making energy use "cheaper" encourages a more active expansion of energy consumers and broader utilization of available resources. In this context, it is interesting to mention the results of the study [100]. In this work, the simplest computer simulation of the evolution of agents capable of possessing different energy strategies is considered. The model is based on fundamental thermodynamic principles, as well as the mechanisms of selection, inheritance, and variability. The work solves the problem of detecting a universal strategy as a result of the selection of possible competing strategies. It is shown that when there is disequilibrium between the environment and the agents, a direction in their evolution arises. At the same time, regardless of the strategy used by each of the agents, the success of the strategy turns out to be closely related to the maximum total dissipation of the agents. Thus, it is not a specific strategy but the tendency to maximize dissipation that is universal in evolution.

Does MEPP not contradict the fact that species with different entropy production coexist on Earth, and why is there not only one winner? A similar question arose earlier regarding Darwin’s theory. The answers here are largely similar. There are winners (leaders in entropy production) for each ecological niche. As follows from MEPP formulation, extremization (selection) occurs each time under existing restrictions, which can be different (due to habitat conditions, for example). Moreover, according to MEPP, extremization occurs at every level of hierarchy, which is why more highly organized species can coexist with less organized ones, as they belong to different levels—just as cells coexist with organisms, for instance.

The ideas of striving for perfection and natural selection, proposed earlier to explain the observed biological evolution, are essentially consequences of MEPP, which underlies the evolution of any non-equilibrium processes in the surrounding world, not being divided into “living and nonliving kingdoms”. MEPP, in turn, is closely related to the entropic measure of time, as was shown earlier in Section 3. As demonstrated above, the view of the developing world using EMT leads to self-evidence of MEPP. Obviously, something similar should be observed when considering evolution: the organisms that win in selection obviously have the greatest duration of existence in the evolutionary sense and therefore, according to Equation (1), they have the greatest specific entropy production.

The author is not aware of any works in which the behavior of dissipation (intrinsic time), depending on extrinsic (astronomical) time in the case of evolutionary selection, is quantitatively studied using experimental material. An indirect indication that this dependence is consistent with Equation (3) are the results of a well-known long-term evolutionary experiment on *Escherichia coli* bacteria [101,102,103]. As was obtained in these experiments, the increase in the rate of bacterial reproduction (and hence entropy production) in comparison with the original, ancestral strain during evolution initially increases sharply, and then slows down, but does not reach a plateau. Being constantly under the same conditions, the experimental bacteria constantly accumulate useful mutations, steadily increase their fitness and, as a result, acquire the ability to use resources for life that they had not previously assimilated. As a consequence, as the authors of [102] note, real reproduction rates cannot be described by hyperbolic models that demonstrate asymptotic growth to some constant value, but it is necessary to use models that demonstrate a weak permanent increase at large times. Obviously, the logarithmic model (3) is precisely one of such models. The studies [101,102,103] inspired the work [104], which examined a simple evolutionary model for developing an algorithm in an agent (predator) to capture a moving prey. The simulation was conducted using a software-emulated Intel 8080 processor. Maximizing the number of captures was chosen as the objective function. This work demonstrates that the logarithmic model (3) is indeed applicable and offers several advantages for describing the observed increase in dissipation during the evolutionary process.

## 6. Conclusions

The review demonstrated that the introduction of intrinsic time based on entropy production provides a unified perspective on a wide range of contemporary problems, spanning from cosmology to developmental biology and evolution. The logarithmic relationship between intrinsic and extrinsic time for developing systems of various natures is very interesting and promising. It is extremely important that the use of EMT allows us to take a new look at the most important modern approaches to explain evolution: natural selection and MEPP. As a result, these classical approaches turn from non-trivial statements into obvious statements. In this regard, we can recall the statement of Josiah Gibbs (1881): “One of the principal objects of theoretical research is to find the point of view from which the subject appears in the greatest simplicity” [105].

Entropic time can only be introduced and is useful for irreversible processes; it loses its meaning when considering processes described by reversible equations of mechanics, electromagnetism, or quantum physics. Real irreversible processes have a beginning and an end, breaking the uniformity of Newtonian time symbolizing eternity and emptiness. Growth and evolution are typical examples of such processes. As a result, entropic time is not an eternal characteristic of a process. On the contrary, it appears with the birth of an irreversible process and disappears with it. As a consequence, the introduced entropic time directly follows the opinion of the ancient Greek philosopher Plato, who believed that true time is a simple negation of eternity.

Entropic time can be introduced for any irreversible process; however, unlike Newtonian time, this time is neither uniform nor a universal measure for all processes. Entropic time is an individual characteristic of each irreversible process. This time is most closely associated with the process. And this has its positive side, since such time allows us to consider the process in the simplest and least distorted natural way. Several examples illustrating this have been provided in the study. The fact that an individual, rather than a general description, gives advantages is known in physics. For instance, when describing spherically symmetric systems, a coordinate system corresponding to the symmetry is used for simplicity, rather than a universal Cartesian coordinate system. Or, for example, when describing the motion of the atmosphere near the Earth, a reference system associated with the rotating Earth allows the least cumbersome mathematical description of the process, despite the occurrence of an additional (Coriolis) force, which is absent when using a universal inertial reference system.

Reversible and universal equations of physics, as is known, not only often lead to the conclusion about the illusory nature and redundancy of one of their variables, time, but also to the conclusion that everything around us is entirely deterministic and predictable. Entropic time associated with some irreversible process, on the contrary, is the most important characteristic of a specific process and, as was shown in this review, this time itself can be a constructor of equations describing the development of this process. Thus, the irreversible process itself, through time, determines its future. Such a solution to the oppressive problem of determinism and the absence of "free will" in the development of irreversible systems is new and intriguing. Here, it is appropriate to recall again the opinion of E. Milne, mentioned in Section 2, that the structure of the system under consideration, including its fundamental concepts, should determine the laws that operate within it. It is important to further explore this possibility using traditional natural scientific methods and philosophically.

## Data Availability

No new data were created or analyzed in this study. Data sharing is not applicable to this article.
